# How Do Neurons Signal Itch?

**DOI:** 10.3389/fmed.2021.643006

**Published:** 2021-03-15

**Authors:** Martin Schmelz

**Affiliations:** Department of Experimental Pain Research, Medical Faculty Mannheim, University of Heidelberg, Mannheim, Germany

**Keywords:** itch theories, temporal pattern, specificity, spatial contrast, pruriceptor, pain

## Abstract

Mechanistic theories of itch are based on neuronal specificity, stimulus intensity, and temporal or spatial discharge patterns. Traditionally, these theories are conceptualized as mutually exclusive, assuming that finding evidence for one theory would exclude the others and could sufficiently explain itch. Current experimental data primarily support the specificity or pattern theory of itch. However, in contrast to an assumed inherent exclusivity, recent results have shown that even within itch-specific pathways in the spinal cord, temporal discharge patterns are important as sustained pruriceptor is required to allow successful transsynaptic signal progression. Also, optogenetic activation of pruriceptors suggest that the combination of neuronal specificity and temporal pattern determines the sensory effect: tonic activation of pruriceptors is required to induce scratching behavior whereas short-lasting stimulation rather causes withdrawal. In addition to the mere duration of discharge, also the temporal pattern or spatial aspects could critically contribute to elicit pruritus instead of pain. Basic neurophysiological studies trying to validate neuronal theories for pruritus in their pure form provide unitary concepts leading from neuronal discharge to the itch sensation. However, the crucial clinical questions have the opposite perspective: which mechanisms explain the chronic itch in a given patient or a given disease? In trying to solve these clinical problems we should not feel bound to the mutual exclusive nature of itch theories, but rather appreciate blending several theories and also accept combinations of itch and pain. Thus, blended versions of itch theories might better suffice for an explanation of chronic itch in patients and will improve the basis for mechanistic treatment options.

## Introduction

**Figure 1:**
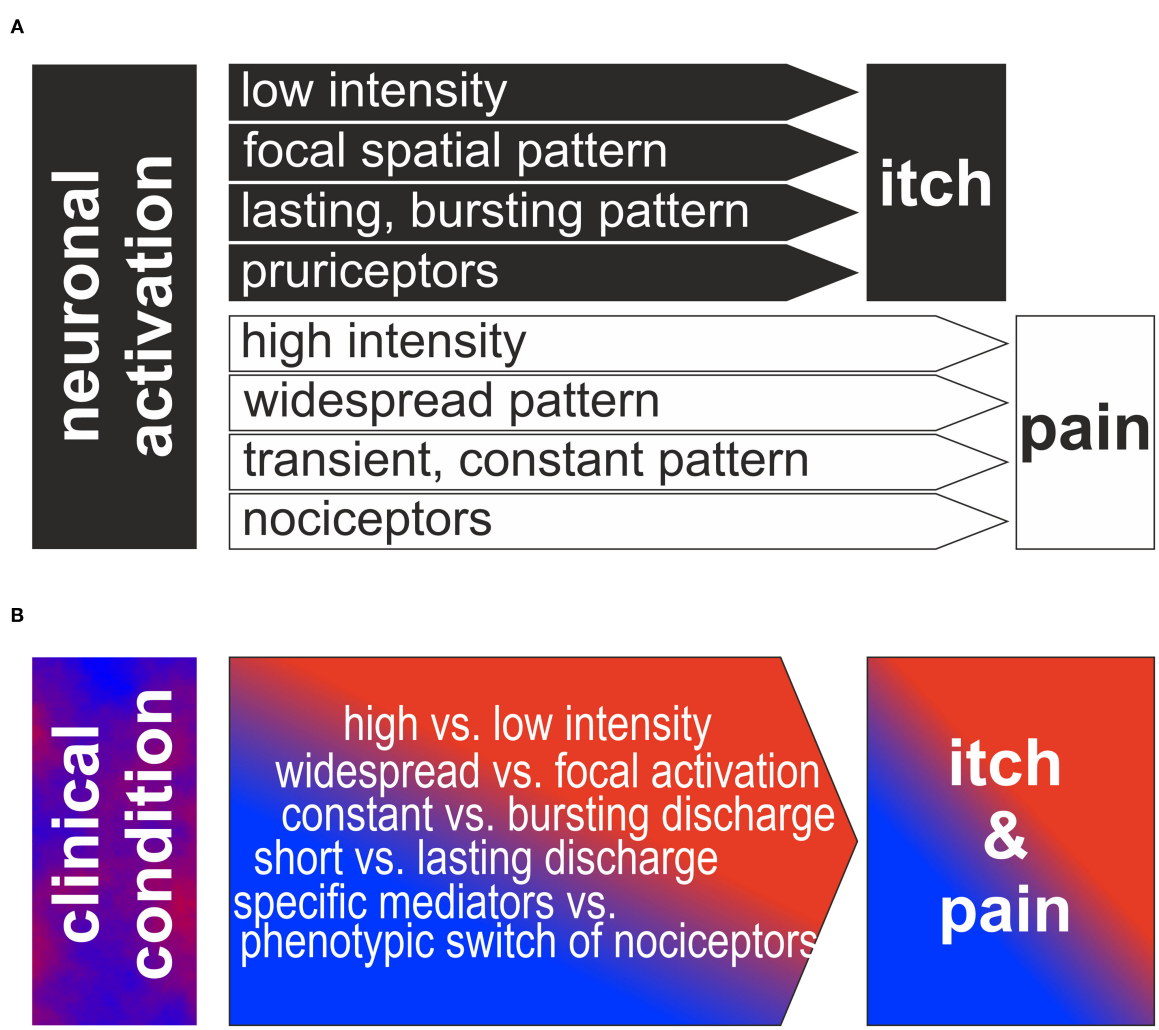
**(A)** Traditional view of neurophysiologic itch theories: Based on low intensity, focal spatial and bursting temporal pattern or to activation of a specific population of pruriceptors itch is generated. In contrast, higher intensity, widespread or constant discharge patterns or the activation of a specific population of nociceptors is generating the sensation of itch. Note, that the different theories are often assumed to be mutually exclusive and binary. **(B)** Reversed perspective is shown: how can the clinical neuronal discharge patterns lead to itch and pain in the patients? We propose that a combination of the different factors intensity, spatial/temporal pattern and specific pathways is required to produce the observed complex combination of clinical itch and pain.

Several neurophysiological theories have been proposed to explain itch based on neuronal specificity (“labeled line”), stimulus intensity, or temporal & spatial discharge patterns (1). Assuming that these theories are mutually exclusive, authors are tempted to generalize their finding when providing evidence for one theory that could sufficiently explain itch (2). Evidence for the “labeled line” processing of itch via histamine sensitive fibers exist for human and cat (2, 3), but not for monkey (4). Clinically, it is obvious that most chronic itch conditions in patients cannot be sufficiently treated with anti-histamines shedding doubts on the clinical importance of a histamine-dependent itch pathway. More recent data from rodents have identified specific non-histaminergic pathways and thus, one might easily switch to a specificity theory of itch based on non-histaminergic pathways. However, it has become obvious that even supposedly itch-specific pathways have complex interactions with pain-processing in the spinal cord “leaky gate.” We might follow up on the traditional neurophysiologic approach that tests certain isolated neuronal activation pattern for their ability to cause the itch sensation (Figure 1A). In this review we will try to change perspective and start with the complex clinical condition (Figure 1B). We focus on the question: how can we explain a complex clinical combination of chronic itch and pain based on the divergent neurophysiological determinants: intensity, pattern and specificity of primary afferent neurons. The complex processing of noxious and pruritic information in the spinal cord (5–7) and more central circuits (8) have been targeted excellently (9–11) and will not be part of this review.

Experimental protocols often are designed along the lines of the intensity-, pattern- or specificity theory of itch suggesting mutual exclusivity. Here we suggest that combinations of the pure theories are providing a framework that might be more adequate to explain clinical itch conditions.

## Classic Theories of Pruritus and Evidence for Interactions Between Them

### Specificity

More than 20 years ago, mechanoinsensitive (“silent”) histamine-sensitive C-nociceptors in human (2) and spino-thalamic projection neurons in the cat (12) have been identified as part of a specific pruritic pathway. More recently, molecular markers of non-histaminergic itch-specific neurons were identified in rodent, such as B-type natriuretic peptide (BNP) (13, 14) and members of the mas-related G-protein receptor family (mrgprA3, C11) (15–17) in primary afferent neurons, but also gastrin releasing peptide (GRP) (18–20) in dorsal horn neurons. Non-histaminergic itch signaling has received major interest when mas-related G-protein coupled receptors (Mrgprs) were identified on presumably itch-related neurons in the mouse, i.e., MrgprA3 (21), MrgprD (22), and MrgprC11 (23). For humans, BAM8-22, an activator of MrgrpC11 (24) and beta-alanine–an activator of MrgprD—provoke itch in humans (22, 25). Chloroquine has often been used in mice to elicit itch-behavior via activation of MrgprA3 (21, 26, 27). Thus, the plethora of new information on pathways and mediators for itch have been found and summarized in excellent reviews (8, 10, 28). All the itch-specific markers and pathways might imply that among the competing itch theories the “labeled line” theory has finally been validated.

However, not necessarily these pruritic mediators are part of a labeled line. In mice, peripheral nerve injuries provoke a broad *de-novo* GRP expression in DRG neurons (29). Thus, upon activation or via spontaneous activity such a “phenotypic switch” might convert nociceptors that typically inhibit itch into neurons that by releasing GRP spinally actively contribute to chronic itch. It is important to note that no direct synaptic contact might be required to get access to the spinal itch pathway, but volume transmission (30) might suffice. Single cell expression patterns have been used to define pruriceptive subpopulations of primary afferent neurons in mouse revealing the key marker proteins Mrgpr A3 (often co-expressing MrgprA1 and MrgprC11), B-type natriuretic peptide / Somatostatin, and MrgprD (31–33). In humans, classification of primary afferent nociceptors is classically based on their sensory characteristics obtained from single fiber recordings (34–38), such as mechanical thresholds and temperature sensitivity (Table 1). Currently, important work is being performed to reduce the translational gaps between rodent and human single cell expression patterns (39, 40), but also to link functional characteristics and expression pattern within a single neuron (41). This ongoing work will provide a much more detailed basis for the differentiation between itch and pain processing neurons and the extent of overlap in mediators and receptors.

**Table 1:** Translational challenges in pruriceptor classification.

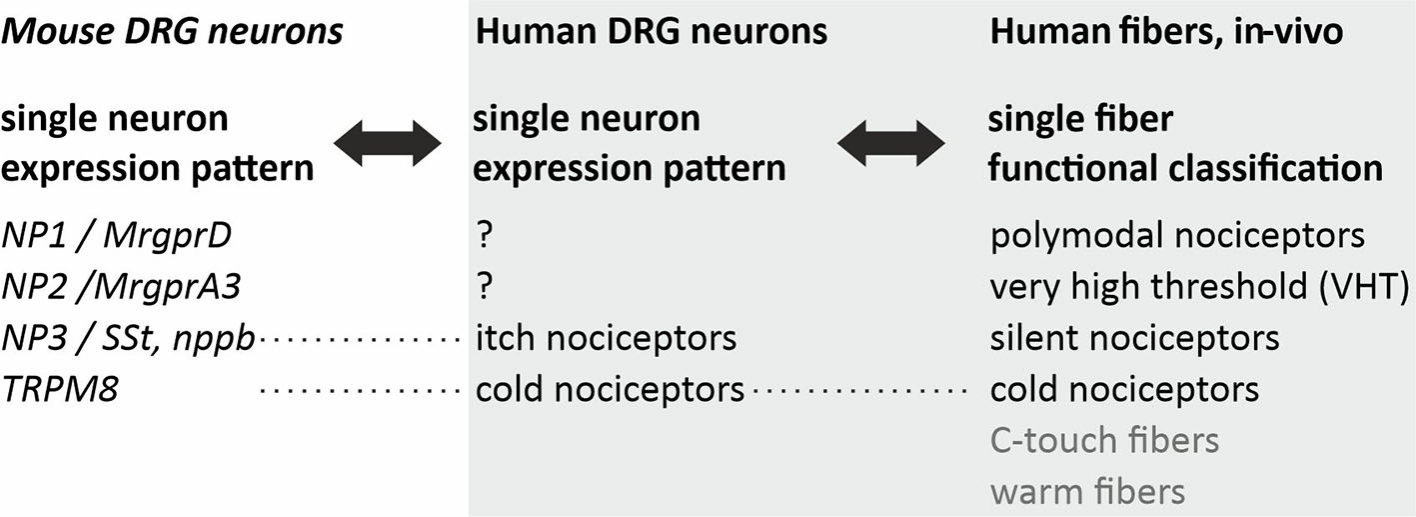

### Temporal and Spatial Pattern

Electrophysiological data from rodents and monkey did not support a “labeled line” for itch (28, 42–44) as no specific subpopulation of itch neurons was found. The results rather support the pattern theory of itch according to which nociceptors can signal itch or pain based on the combination of activated fibers resulting in a population coding (1) or based on specific discharge patterns that might differentiate between itch and pain (1).

**Figure 2:**
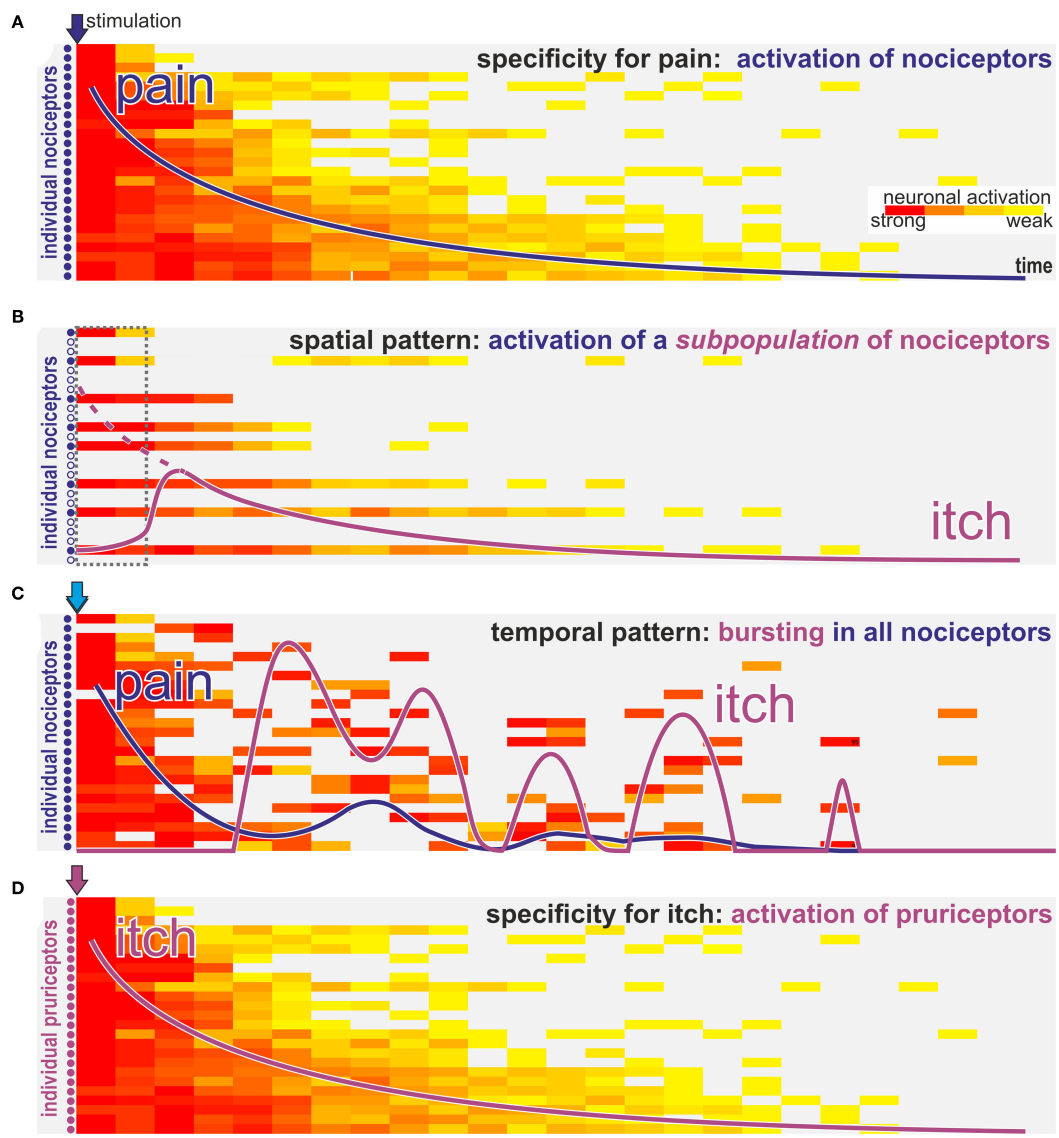
How can nociceptors signal itch? Schematic view of activation intensity (weak to strong indicated by yellow to red color) of single neurons over time after stimulation with algogens (blue arrows **A–C**) or a pruritogen (purple arrow **D**). **(A)** Classic activation of nociceptors by stimulation with a pure algogen: activation of nociceptors in a decrescendo pattern is felt as gradually declining pain sensation (blue line). **(B)** In case of algogens that provoke lasting activation only of a selective subpopulation of nociceptors the activation pattern resembles a “spatial contrast” with silent and highly active nociceptors innervating the same stimulation site. Such a spatial activation pattern is interpreted on spinal level as itch. However, the itch sensation (purple line) will start with some delay required to allow spinally released gastrin releasing peptide to allow activation of the itch pathway (dotted interval in gray; dotted purple line indicates the magnitude of the peripheral input). **(C)** For algogens that provoke prolonged bursting activation patterns there may be an initial phasic activation of all nociceptors leading to a transient pain sensation (blue line). However, in the ensuing phase randomly bursting neurons will stochastically provoke a pattern that resembles the “spatial contrast” shown in B and that is interpreted as itch (purple line) on a spinal level. **(D)** Classic pruritogens are activating specific pruriceptors that will provoke an itch sensation (purple line) according to the specificity theory.

Very focal activation of nociceptors in the skin has been suggested as a spatial discharge pattern that could explain itch without a “labeled line:” noxious stimulation that is directed only to few cells within the epidermis elicits itch in human skin (45), even when the stimulus is the algogen capsaicin known to activate most human C-nociceptors (46). It has been suggested that local activation of only few epidermal nociceptors can cause itch by a “mismatch signal” (47) or “spatial contrast” (48), provided by few activated and many non-activated nociceptive endings innervating the same skin site, for example by a minute glass wool fiber. Such a discharge pattern indicates that a noxious event is minute and localized within the epidermis. Teleologically, one might conclude that scratching off a part of the epidermis is an adequate response as it will eliminate the threat in this situation. Moreover, scratching will elicit a consistent response of all mechanosensitive polymodal nociceptors innervating this skin and might thereby terminate the “spatial contrast” itch pattern. As shown in Figure 2 such a spatial contrast can not only result from a very focal stimulation in the epidermis, but may also result from strong activation of only a subpopulation of the nociceptors at the stimulation site. Importantly, this activated subpopulation not necessarily belongs to an itch-selective class of nociceptors, it is rather the resulting “spatial contrast” of their response that is responsible for the interpretation as itch on a spinal level.

When the temporal aspects of neuronal discharge are considered, primary afferents activated by the pruritogen histamine are characterized by lower frequencies as compared to discharges to the algogen capsaicin and periods of bursting have longer intervals (49). However, this difference does not allow a clear separation. New data have found differential temporal discharge pattern to heat stimuli between potential primary afferents linked to pain or itch processing: polymodal nociceptors were differentiated according to their response to a step-like noxious heat stimulus into quick (“QC”) or delayed/slow fibers (“SC”) in the monkey (25). C-nociceptors with immediate phasic heat responses were particularly responsive to the pruritogen β-alanine, suggesting that the QC/SC classification might be helpful to characterize a pruriceptive subpopulation (25). Beyond such a characterization, it does is not obvious how this acute response pattern might be linked to a differential signal in itch or pain processing.

As noted above another important temporal aspect is duration and intensity of the discharge: longer lasting activation and higher frequency of GRP-positive neurons were required to finally allow transsynaptic activation of the tertiary GRP-receptor neurons (30). Thus, even when stimulating the itch-specific pathway via optogenetic stimulation of GRP-positive neurons single pulses were inefficient and repetitive lasting stimulation was required (30). Such a temporal pattern of delayed starting of itch after pruritogen application resonates with the psychophysics observed in humans (2). Moreover, in accordance with these considerations optogenetic studies investigating scratching upon specific stimulation generally use prolonged activation periods for example to show disinhibition via dynorphin spinal neurons (30 min at 20 Hz) (50).

### Intensity

According to the intensity hypothesis of itch (51) low level activation of unspecific nociceptors would induce pruritus, whereas higher discharge frequencies would provoke pain. Clinically, impairment of pain processing in neuropathy allowed to provoke itch in some patients (52): in patients with impaired nociception, but intact touch system painful stimuli were felt as itch whereas more specific stimulation using “itch powder” was ineffective (52). Thus, the authors concluded that low intensity of slow nociceptive input provokes itch, whereas increasing the intensity of noxious input by scratching would inhibit itch. Also, the observation that intradermal application of high concentrations of some pruritogens, e.g., histamine, may cause pain seems to be consistent with this hypothesis. However, application of low concentrations of algogens generally does not cause itch, just less intense pain (1). Furthermore, intraneural electrical microstimulation of human afferent C-fibers usually induces pain and, very rarely, pruritus. Increasing the stimulation frequency of intraneural microstimulation enhances the intensity of pain or itch, but no switch from pruritus to pain has been observed. Likewise, a decrease of stimulation frequency at a nerve site where pain has been elicited, decreases the magnitude of pain; but at no point does it induce itch (53).

In summary, the pure intensity theory according to which pruritus is gradually converted to itch upon increasing stimulation without change of quality can be regarded as rejected. Yet, as will be discussed below, there might also be indirect evidence for “unmasking” of itch when the pain input is impaired or treated. Thus, it might not be adequate to leave out all possible aspects of the intensity theory.

## Combinations of Temporal and Spatial Patterns Interacting With Specific Pathway

Basic itch mechanisms are generally discussed in their “pure” form. However, as shown above, there is evidence that itch processing—in particular under pathophysiologic conditions–may combine elements from different basic theories traditionally regarded as mutually exclusive (see Figure 1).

Under experimental conditions, such combinations may be required to consistently explain experimental data: activation of subpopulations of C-afferents with traditional algogens such as capsaicin or endothelin or even populations, such as MrgprA3 positive nociceptors (54, 55), might generate itch not only via the assumed specificity, but will also activate only a subset of nociceptors innervating a given skin site. Broad activation of nociceptors frequency-dependently causes pain (Figure 2A). However, when only a subpopulation of these nociceptors is activated the resulting discharge pattern resembles a “spatial contrast” pattern (Figure 2B) discussed above. Assuming that such an itch signal might require some time to “open the spinal gate” (30) the itch sensation might start with a corresponding delay (dotted box in Figure 2B). Thus, it is important to note, that the term “spatial contrast” is not limited to highly localized stimulation within the epidermis, but can also result from activating only a certain subpopulation of nociceptors. This is even true if this population does not belong to the dedicated specific pruriceptors shown in Figure 2D. Interestingly, even the temporal discharge pattern of single nociceptors may generate such spatial contrast: when chemical stimulations induce discharge in a burst pattern the initial broad response may occur in a synchronized fashion and thereby mainly induced pain (Figure 2C). However, as bursts become less frequent over time there will be stochastically periods in which only few fibers burst in synchrony whereas neighboring fibers are silent thereby again creating the pattern of spatial contrast (Figure 2C).

Under pathophysiological conditions such as neuropathy innervation density is reduced and local stimulation may lead to a spatial contrast based on only few remaining fibers resembling historical reports on itch induced by normally painful stimulation in patients with impaired nociceptors (52). Moreover, another element of the old intensity theory might still be useful under clinical conditions: Assuming that a high number of spontaneously active nociceptors signal pain in a given patient; upon reduction of this number, for example by analgesic therapy, the chances to create a spatial contrast pattern by the still active nociceptors is increasing. This would represent a combination of a spatial contrast theory and the old intensity theory. Interestingly, clinical observations indeed support such a development: in patients with postherpetic neuralgia, resolving pain may be combined with an increase in itch (56, 57). Thus, based on defined experimental models, we have successfully developed basic theories that can explain differentiation between itch and pain based on specificity in a “labeled line” or the discharge pattern in its temporal or spatial expression (Figure 2, lower part). These approaches provide us with powerful tools when trying to explain clinical neuropathic itch. However, rather than assuming that in pathological conditions there is a mutually exclusive explanation for itch purely based on one theory of itch, we rather might adapt our conceptual framework and include mechanisms that borrow aspects from different theories.

In summary, discharge of subpopulations of primary afferent sensory neurons can contribute to the itch generation via their specific spinal connection to pruriceptive pathways and via their spatial and temporal pattern including to some extent also the intensity of discharge. Thus, even on the relatively simple level of primary afferents we find a clear overlap between traditionally separated and mutually exclusive concepts of itch (see Figure 1A). Therefore, it appears adequate to allow combinations of itch theories for the explanation of complex clinical itch conditions (see Figure 1B). However, this rather theoretic aspect does not sufficiently capture the complexity inherent in the clinical setting: pathophysiologic changes linked to chronic itch conditions may affect crucial characteristics of peripheral and central neurons. These changes my affect even well-established markers such as gastrin releasing peptide that has been shown to be broadly upregulated in primary afferent neurons after axotomy (29). In addition to such specific changes of pruriceptive processing, there are broader consequences of neuropathy that impinge on itch processing, such as partial denervation of nociceptors, expression pattern changes, Wallerian degeneration & regeneration, and glial activation. These processes can fundamentally change neuronal excitability leading to modified discharge patterns and spontaneous activity that will generate an even more complex scenario. Chronic inflammation can lead to corresponding excitability changes with and without concomitant neuropathic or neuroplastic changes and therefore even increases the complexity under clinical conditions. Therefore, our ability to predict sensory effects in patients with neuropathy or chronic inflammation differentiating between itch and pain are limited.

### Perspectives and Implications

*Implications for basic experimental approaches*. Current experimental data primarily support the specificity and pattern theory of itch. However, recent results have shown that temporal discharge patterns have crucial effects (49) even within itch-specific pathways in the spinal cord: sustained peripheral input via pruriceptors is required to enable successful transsynaptic signal progression in the spinal cord (30). Optogenetic techniques have enabled unmatched temporal control of specific classes of primary afferents (50, 58–61). Again, neuronal specificity and temporal pattern interacted: tonic activation of pruriceptors is required to induce scratching behavior whereas short-lasting stimulation might be ineffective or may even cause withdrawal (30). Thus, the implications of temporal patterns need to be considered even when the experimental approach is focused on an itch-specific pathway, i.e., upon optogenetic stimulation of pruriceptors it is important to compare responses to acute phasic stimulation and tonic discharge. Moreover, the spatial aspect of activation might play a role even for specific activation using optogenetic stimulation in particular for transcutaneous stimulus when the limited penetration depth of blue light is taken into consideration.

When analyzing the discharge of primary afferents we usually analyze area under the curve and maximum discharge frequency. However, considering the importance of other temporal patterns such as lasting and bursting discharge we might expand our analysis accordingly as bursting discharge patterns of nociceptors with long silent periods in between can also generate some kind of spatial contrast pattern thereby merging temporal and spatial contrast theory. On the other hand, synchronous input of high discharge of many nociceptors will cause pain thereby incorporating aspects of the intensity theory to the overall outcome.

*Implications for clinical approaches:* The “phenotypic switch” of nociceptors that start to express GRP after axotomy (29) not only adds to the controversy about the role of this peptide in primary afferents (62, 63), but may have major implications for clinical pruritus. Beyond the direct involvement of GRP in the generation of pruritus it may render the spinal cord more susceptible for evoked pruritus: in particular acute electrical stimulation may provoke mainly pain in healthy volunteers. However, in chronic itch patients it might also evoke itch (64) suggesting that the spinal cord processing of itch has been facilitated in these patients potentially be previous release of GRP. For experimental stimulation protocols therefore phasic and tonic stimulation paradigms and possible carry-over effects by spinal GRP release need to be considered.

Animal models have been optimized to separately assess intensity of itch and pain, for example via scratching vs. wiping behavior upon injection into the cheek has been successfully established in rodents (65). However, even under these experimental conditions these models can also elicit a combination of itch- and pain-like behaviors (65, 66) suggestive of a mixed sensation. Indeed, patients with small fiber neuropathy often report concomitant itch and pain sensations (67) such as “burning itch” or “itching sting.” Rather than separating itch and pain according to the involved discipline (pain for anesthesiologists/neurologists vs. itch for dermatologists) we need to further our translational efforts not only between basic researchers and clinical scientists, but also between pain and itch specialists.

## Author Contributions

The author confirms being the sole contributor of this work and has approved it for publication.

## Conflict of Interest

The author declares that the research was conducted in the absence of any commercial or financial relationships that could be construed as a potential conflict of interest.
